# Untargeted Metabolomic Analyses and Antilipidemic Effects of Citrus Physiological Premature Fruit Drop

**DOI:** 10.3390/ijms25031876

**Published:** 2024-02-04

**Authors:** Chao Wang, Mingfang Peng, Zhipeng Gao, Qi Han, Fuhua Fu, Gaoyang Li, Donglin Su, Lvhong Huang, Jiajing Guo, Yang Shan

**Affiliations:** 1 Longping Branch, College of Biology, Hunan University, Changsha 410125, China; 2 Hunan Agriculture Product Processing Institute, Dongting Laboratory, Hunan Provincial Key Laboratory of Fruits & Vegetables Storage, Processing, Quality and Safety, Hunan Academy of Agricultural Sciences, Changsha 410125, China; 3 Fisheries College, Hunan Agricultural University, Changsha 410128, China; 4 College of Animal Science and Technology, Hunan Agricultural University, Changsha 410128, China

**Keywords:** citrus premature fruit drop, bioactive components, antioxidant, untargeted metabolomics, antilipidemic effect

## Abstract

Increasingly globally prevalent obesity and related metabolic disorders have underscored the demand for safe and natural therapeutic approaches, given the limitations of weight loss drugs and surgeries. This study compared the phytochemical composition and antioxidant activity of five different varieties of citrus physiological premature fruit drop (CPFD). Untargeted metabolomics was employed to identify variations in metabolites among different CPFDs, and their antilipidemic effects in vitro were assessed. The results showed that *Citrus aurantium* L. ‘*Daidai*’ physiological premature fruit drop (DDPD) and *Citrus aurantium* ‘*Changshan-huyou*’ physiological premature fruit drop (HYPD) exhibited higher levels of phytochemicals and stronger antioxidant activity. There were 97 differential metabolites identified in DDPD and HYPD, including phenylpropanoids, flavonoids, alkaloids, organic acids, terpenes, and lipids. Additionally, DDPD and HYPD demonstrated potential antilipidemic effects against oleic acid (OA)-induced steatosis in HepG2 hepatocytes and 3T3-L1 adipocytes. In conclusion, our findings reveal the outstanding antioxidant activity and antilipidemic effects of CPFD, indicating its potential use as a natural antioxidant and health supplement and promoting the high-value utilization of this resource.

## 1. Introduction

Obesity has become a major public health concern due to the drastic lifestyle changes of the global population. It is estimated that approximately 500 million people worldwide suffered from metabolic disorders and obesity in 2020 [1]. As obesity rates continue to rise, more people risk developing diseases such as diabetes, cardiovascular disease, and fatty liver [2]. Reactive oxygen species (ROS) are metabolism byproducts that give rise to cellular oxidative stress when in excess [3], causing obesity-related complications [4]. ROS alter the concentrations of molecules involved in inflammation, particularly adipocytes. Changes in the size and abundance of adipocytes promote fat generation and adipogenesis, thus stimulating pre-adipocyte differentiation into mature adipocytes [5,6]. Non-alcoholic fatty liver disease (NAFLD) is associated with obesity [7]. This emerging chronic liver disease disrupts lipid metabolism and increases the synthesis and accumulation of liver fat, resulting in various liver dysfunctions [8]. Therefore, reducing oxidative stress and inhibiting fat generation are promising treatment options for obesity prevention.

Citrus is a plant genus that belongs to the *Rutaceae* family, comprising various species such as *Citrus sinensis* (orange), *Citrus reticulata* (mandarin), *Citrus aurantifolia* (lime), *Citrus limon* (lemon), *Citrus paradisi* (grapefruit), *Citrus bergamia* (bergamot), *Citrus junos* (yuzu), and *Citrus japonica* (kumquat) [9]. Citrus plants are extensively cultivated in tropical and subtropical regions across the globe, making them one of the foremost fruit crops worldwide. Citrus fruits are rich in active substances such as carotenoids, essential oils, pectin, flavonoids, and limonoids [10,11], which contribute to their antioxidant, anti-allergic, anti-inflammatory, anticancer, blood pressure regulation, and lipid reduction properties [12,13]. According to the Food and Agriculture Organization (FAO), the global citrus cultivation area spanned 1.01 million ha in 2020 [14]. Generally, the fruit-setting rate of citrus is very low (3–5%), leading to high premature fruit drop [15].

Despite various surgical and pharmacological treatments, no risk-free and effective weight management therapy is currently available. Changes in lifestyle, diet, and reduction in sedentary behavior are considered the best options for obesity prevention [16]. Phytotherapy targets health issues by using plant-derived drugs. Additionally, natural plant supplements lead to significant weight loss and improved health by neutralizing ROS. Plant-based products are alternatives to weight management due to the rich active chemical substances, including polyphenols, flavonoids, carotenoids, and catechins. Citrus physiological premature fruit drop (CPFD) is a normal phenomenon during the fruit development process, which is distinct from fruit drop caused by storms, diseases, and pests. Previous studies have shown that the active plant components in dropped citrus fruits are higher than those in mature fruits [17]. However, the current research on the utilization of citrus byproducts is mainly focused on processing residual materials from mature fruits, while studies on CPFD utilization are scarce. Currently, only a small proportion of CPFD is collected and used for traditional Chinese medicine, while most are considered agricultural waste. Therefore, repurposing CPFD can reduce waste and environmental pollution. Moreover, this agricultural byproduct is an attractive source of phytochemicals for human dietary supplements. 

Increasing evidence suggests that citrus fruits are rich in flavonoids and good regulators of lipid metabolism. In this study, we compared the phytochemical content and antioxidant activity of physiological premature fruit drop and mature fruits of five different citrus cultivars. Subsequently, untargeted metabolomic analysis was performed on two citrus cultivars with higher phytochemical content and stronger antioxidant capacity to further explore their differential metabolites. In addition, we investigated the antilipidemic effect of selected CPFD on HepG2 and 3T3-L1 cells.

## 2. Results

### 2.1. Differences in Citrus Fruits Chemical Composition

**Figure 1:**
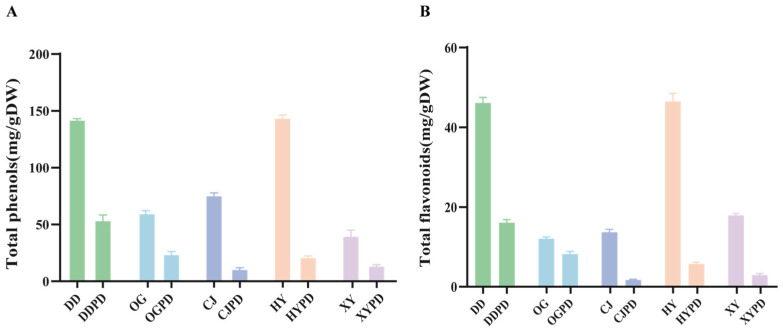
Differences in citrus fruits’ chemical composition. (**A**) The total polyphenol content in CPFDs and citrus mature fruits. (**B**) The total flavonoid content in CPFDs and citrus mature fruits. Error bars are expressed as means ± standard deviation (SD). CPFD, citrus physiological premature fruit drop; DD, *Citrus aurantium* L. ‘*Daidai*’ mature fruit drop; DDPD, *Citrus aurantium* L. ‘*Daidai*’ physiological premature fruit drop; OG, *Citrus reticulata* cv. *Suavissima Ougan* mature fruit drop; OGPD, *Citrus reticulata* cv. *Suavissima Ougan* physiological premature fruit drop; CJ, *Citrus reticulate* ‘*Blanco*’ mature fruit drop; CJPD, *Citrus reticulate* ‘*Blanco*’ physiological premature fruit drop; HY, *Citrus aurantium* ‘*Changshan-huyou*’ mature fruit drop; HYPD, *Citrus aurantium* ‘*Changshan-huyou*’ physiological premature fruit drop; XY, *Citrus maxima* (*Burm.*) *Merr.* cv. *Jiangyong Yu* mature fruit drop; XYPD, *Citrus maxima* (*Burm.*) *Merr.* cv. *Jiangyong Yu* physiological premature fruit drop.

Polyphenols and flavonoids are the primary active components in citrus fruits, which have been reported to be beneficial for health [18]. The total phenolic and total flavonoid contents were determined in this study to evaluate the active ingredients of five selected citrus varieties. The study findings demonstrated that the polyphenol content was highest in *Citrus aurantium* ‘*Changshan-huyou*’ physiological premature fruit drop (HYPD) (144.00 ± 0.95 mg/gDW), followed by *Citrus aurantium* L. ‘*Daidai*’ physiological premature fruit drop (DDPD) (142.27 ± 0.68 mg/gDW), *Citrus reticulate* ‘*Blanco*’ physiological premature fruit drop (CJPD) (75.67 ± 1.28 mg/gDW), *Citrus reticulata* cv. *Suavissima Ougan* physiological premature fruit drop (OGPD) (59.76 ± 1.69 mg/gDW), and *Citrus maxima* (*Burm.*) *Merr.* cv. *Jiangyong Yu* physiological premature fruit drop (XYPD) (40.07 ± 4.86 mg/gDW) among CPFDs (Figure 1A). Similarly, HYPD recorded the highest total flavonoid content (46.76 ± 1.23 mg/gDW), followed by DDPD (46.38 ± 0.91 mg/gDW), XYPD (18.18 ± 0.30 mg/gDW), CJPD (13.92 ± 0.36 mg/gDW), and OGPD (12.32 ± 0.16 mg/gDW) (Figure 1B). Notably, the total phenolic and flavonoid contents in CPFD were higher than those in mature fruits. For instance, HYPD total phenolic and flavonoid contents were 6.78 and 7.79 times higher than those in mature fruits, respectively. These results demonstrate that the bioactive components in citrus fruits decrease gradually with maturity.

### 2.2. Antioxidant Activity in Citrus Fruits Extract

**Figure 2:**
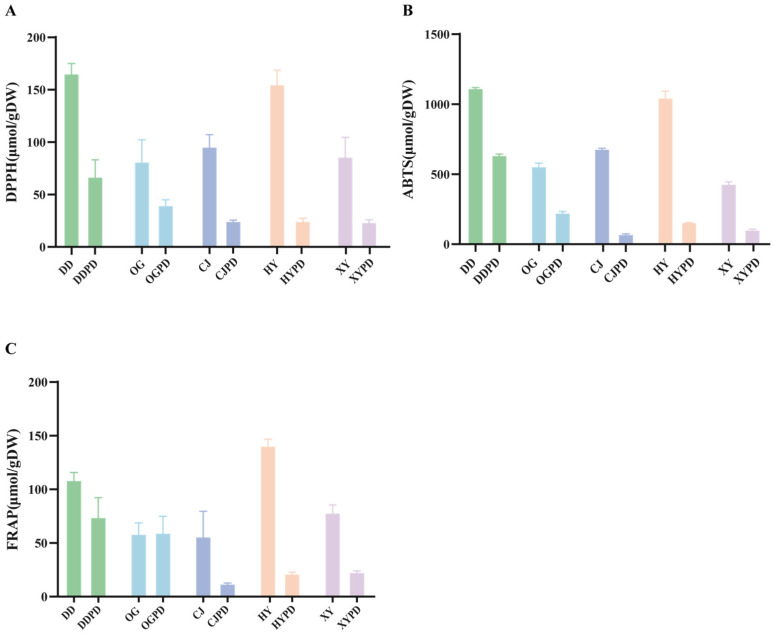
Antioxidant activity in citrus fruit extract. (**A**) Results based on DPPH radical scavenging ability. (**B**) ABTS•^+^ radical scavenging ability. (**C**) Ferric reducing antioxidant power. Error bars are expressed as means ± SD. DPPH, 2,2-diphenyl-1-picrylhydrazyl; ABTS, 2,2-azino-bis(3-ethylbenzothiazodine-6-sulfonic acid); FRAP, ferric reducing antioxidant power; DD, *Citrus aurantium* L. ‘*Daidai*’ mature fruit drop; DDPD, *Citrus aurantium* L. ‘*Daidai*’ physiological premature fruit drop; OG, *Citrus reticulata* cv. *Suavissima Ougan* mature fruit drop; OGPD, *Citrus reticulata* cv. *Suavissima Ougan* physiological premature fruit drop; CJ, *Citrus reticulate* ‘*Blanco*’ mature fruit drop; CJPD, *Citrus reticulate* ‘*Blanco*’ physiological premature fruit drop; HY, *Citrus aurantium* ‘*Changshan-huyou*’ mature fruit drop; HYPD, *Citrus aurantium* ‘*Changshan-huyou*’ physiological premature fruit drop; XY, *Citrus maxima* (*Burm.*) *Merr.* cv. *Jiangyong Yu* mature fruit drop; XYPD, *Citrus maxima* (Burm.) *Merr.* cv. *Jiangyong Yu* physiological premature fruit drop.

Previous research reported that citrus fruits contain active compounds with strong antioxidant properties, and their antioxidant activities vary at different maturity stages [19]. In this study, the 2,2-diphenyl-1-picrylhydrazyl (DPPH) and 2,2-azino-bis(3-ethylbenzothiazodine-6-sulfonic acid) (ABTS) radical scavenging assays and ferric reducing antioxidant power (FRAP) were carried out to assess the antioxidant activity of citrus fruits at premature and mature states. The findings demonstrate that all fruits at the premature stage possess substantial antioxidant capabilities. According to the DPPH radical scavenging activity, the antioxidant values of DDPD, OGPD, CJPD, HYPD, and XYPD were 165.42 ±11.40, 81.23 ± 24.58, 95.61 ± 17.77, 156.14 ± 11.28, and 85.27 ± 18.86 μmol/gDW, respectively (Figure 2A). The DDPD and HYPD exhibited the highest DPPH radical scavenging activity but were not significantly different from one another. Additionally, DDPD and HYPD displayed notable ABTS·+ radical scavenging activity, consistent with the DPPH assay results (Figure 2B). The DDPD (1114.35 ± 5.65 μmol/gDW) exhibited the best ABTS scavenging activity among the CPFD, followed by HYPD (1046.79 ± 54.82 μmol/gDW), OGPD (681.28 ± 3.25 μmol/gDW), CJPD (554.88 ± 27.10 μmol/gDW), and XYPD (430.67 ± 19.94 μmol/gDW). Conversely, HYPD exhibited superior reducing power compared to DDPD (Figure 2C), indicating the potential differences in the underlying mechanisms between different detection methods. Notably, all mature fruits exhibited lower antioxidant activity than CPFDs, especially DDPD and HYPD. This further confirms the presence of a richer phytochemical composition in CPFDs compared to the mature fruits.

### 2.3. Untargeted Metabolomic Analysis of CPFDs

Based on the total phenolic and flavonoid contents and antioxidant activity of CPFD and mature fruit samples of five citrus species, the HYPD and DDPD samples exhibited the best performance for all assessments and, thus, were selected for further analysis. Metabolites in HYPD and DDPD samples obtained 779 features in positive ion mode (ESI+) and 440 features in negative ion mode (ESI−) (Supplementary Materials). Using more metabolic features in chemometric analysis can yield more reliable results in untargeted metabolomics analysis. In this study, principal component analysis (PCA) was introduced to the processed data matrix to reduce data dimensionality and enhance data interpretability.

#### 2.3.1. Principal Component Analysis of CPFD

**Figure 3:**
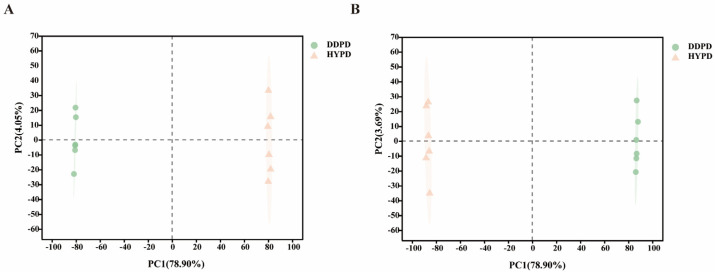
Principal component analysis of CPFD. (**A**) Positive ESI mode. (**B**) Negative ESI mode. CPFD, citrus physiological premature fruit drop; DDPD, *Citrus aurantium* L. ‘*Daidai*’ physiological premature fruit drop; HYPD, *Citrus aurantium* ‘*Changshan-huyou*’ physiological premature fruit drop.

Unsupervised PCA was employed to observe the overall clustering and distribution trends between HYPD and DDPD. The observed substantial dispersion between HYPD and DDPD suggests noteworthy variances in metabolite profiles. In the ESI+ model, axis 1 (PCoA1) indicated 78.90% of total variability, while axis 2 (PCoA2) demonstrated 4.05% of variability (Figure 3A). In the ESI− model, axis 1 also showed 78.90% of total variability compared to only 3.69% in axis 2 (Figure 3B).

#### 2.3.2. Screening and Identification of Candidate Differential Metabolites

Identifying candidate differential metabolites in untargeted metabolomics can be challenging, thus requiring accurate molecular weights, retention times, and ion fragments. In order to identify differentially expressed metabolites between HYPD and DDPD, we utilized the partial least squares discriminant analysis (PLS-DA) model to calculate the variable importance for projection (VIP) values. Additionally, to ensure higher accuracy and reliability in the structural elucidation of metabolites, we selected differential metabolites based on the criteria of Fragmentation Score > 50, VIP > 1.00, and *p* < 0.05. In this study, 87 compounds were identified in ESI+ mode and 10 compounds in ESI- mode as differential metabolites (Fragmentation Score > 50) (Table 1). Among the 97 compounds, DDPD upregulated 41 and downregulated 56 compounds compared to HYPD. These candidate differential metabolites comprised 4 phenylpropanoids, 7 flavonoids, 7 alkaloids, 10 organic acids, 4 terpenes, 27 lipids, and 38 other metabolites.

A. Phenylpropanoids

**Figure 4:**
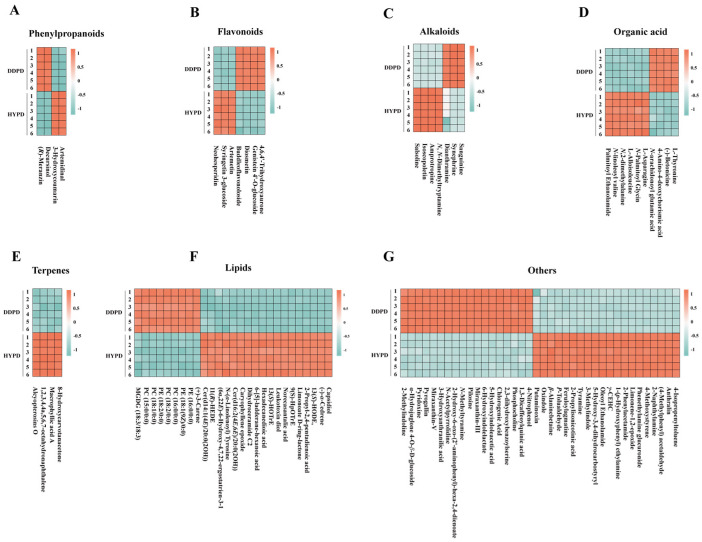
Heatmap showing levels among 97 differentially expressed metabolites in DDPD and HYFD. (**A**) Phenylpropanoids. (**B**) Flavonoids. (**C**) Alkaloids. (**D**) Organic acids. (**E**) Terpenes. (**F**) Lipids. (**G**) Other metabolites. DDPD, *Citrus aurantium* L. ‘*Daidai*’ physiological premature fruit drop; HYPD, *Citrus aurantium* ‘*Changshan-huyou*’ physiological premature fruit drop.

Four phenylpropanoids were identified in DDFD and HYFD, namely (*R*)-meranzin, decursinol, 3-hydroxycoumarin, and artemidinal (Figure 4A). Among them, (*R*)-meranzin and decursinol showed higher levels in DDFD, while 3-hydroxycoumarin and artemidinal showed higher levels in HYFD.

B. Flavonoids

The results revealed the presence of seven significantly expressed flavonoid compounds in DDFD and HYFD (Figure 4B). These include buddleoflavonoloside, genistein 4′-O-glucoside, 4,6,4′-trihydroxyaurone, artemetin, syringetin 3-glucoside, diosmetin, and neohesperidin. Among them, artemetin, syringetin 3-glucoside, and neohesperidin exhibited higher levels in HYPD, while the remaining four compounds showed high expression in DDPD. It is worth noting that diosmetin and neohesperidin are two typical flavonoid compounds found in citrus.

C. Alkaloids

Seven alkaloids were identified in DDFD and HYFD, namely salsoline, isoscopoletin, amprotropine, dianthramine, synephrine, *N*, *N*-dimethyltryptamine, and sanguinine (Figure 4C). Among them, synephrine, sanguinine and dianthramine had higher contents in DDPD, while salsoline, isoscopoletin, amprotropine, *N*, *N*-dimethyltryptamine, and were present in higher amounts in HYFD. It is noteworthy that synephrine is widely present in the citrus fruit peel and belongs to a class of alkaloids [20].

D. Organic acid

A total of ten compounds were identified in this group (Figure 4D). Among them, four compounds showed high expression in DDPD, including 4-amino-4-deoxychorismic acid, (−)-betonicine, L-thyronine, and *N*-arachidonoyl glutamic acid. *N*-linoleoyl valine, L-asparagine, *N*,2-dimethylalanine, L-alloisoleucine, palmitoyl ethanolamide, and *N*-palmitoyl glycine exhibited high expression in HYPD.

E. Terpenes

The identification of 2-Isopropenyl-4a,8-dimethyl-1,2,3,4,4a,5,6,7-octahydronaphthalene, 8-hydroxycarvotanacetone, alcyopterosins O, and macrophyllic acid a was accomplished in both DDPD and HYPD (Figure 4E). Interestingly, compared to DDPD, these four terpenoids were found to be more abundant in the terpenoid fraction of HYPD, implying a higher content of terpenoids in HYPD.

F. Lipids

The results indicated that a total of 27 compounds were identified in this group, among which 9 were highly expressed in DDPD, including PC (16:0/0:0), (+)-3-carene, PC (15:0/0:0), PC (18:1/0:0), PE (16:0/0:0), PE (18:1(9*Z*)/0:0), MGDG (18:3/18:3), PC (18:2/0:0), and PE (18:2/0:0) (Figure 4F). Meanwhile, 18 compounds, namely2-propyl-2,4-pentadienoic acid, caryophyllene epoxide, leukotoxin diol, (6*α*,22*E*)-6-hydroxy-4,7,22-ergostatrien-3-1, dihydroceramide c2, (−)-*α*-cedrene, 11(*R*)-HEDE, cer(d16:2(4*E*,6*E*)/20:0(2OH)), 9(*S*)-HpOTrE, limonoate D-ring-lactone, capsidiol, norecasantalic acid, cer(d14:1(4*E*)/20:0(2OH)), 13(*S*)-HODE, 13(*S*)-HOTrE, *N*-(*α*-linolenoyl) tyrosine, 6-[5]-ladderane-hexanoic acid, and hexadecanedioic acid, showed higher levels in HYPD. This also suggested that there are higher levels of lipids in HYPD compared to DDPD.

G. Others

A total of 38 compounds were identified in this group (Figure 4G). Among them, 18 compounds showed high expression levels in DDPD, including 2-methylindoline, *N*-methyltyramine, 2,3-dihydroxybenzoylserine, 4-nitrophenol, pyridoxine, 6-hydroxyindolelactate, pilosine, 1,3-dicaffeoylquinic acid, phosphocholine, *N*-acetylpyrrolidine, 2-hydroxy-6-oxo-(2′-aminophenyl)-hexa-2,4-dienoate, miraxanthin-III, miraxanthin-V, *β*-alaninebetaine, *α*-hydrojuglone 4-*O*-*β*-d-glucoside, 5-hydroxyindoleacetic acid, chlorogenic acid, pyrogallin, and 3-hydroxyanthranilic acid. On the other hand, 20 compounds exhibited high expression levels in HYPD, namely feruloylagmatine, anthralin, tyramine, 1-(*p*-hydroxyphenyl) ethylamine, putaminoxin, oxindole, (4-methylphenyl) acetaldehyde, 2-phenylacetamide, 3-methylindole, limonene-1,2-epoxide, 4-methoxystyrene, phenethylamine glucuronide, 2-naphthylamine, *p*-tolualdehyde, oleoyl ethanolamide, 5-hydroxy-3,4-dihydrocarbostyryl, 2-propylisonicotinic acid, *γ*-CEHC, and 4-isopropenyltoluene.

Based on the above results, it can be observed that two types of citrus premature fruit drop have distinct metabolomic features with respect to their physiological fruit drop. HYFD is rich in terpenoids and lipids, whereas DDPD and HYFD have similar levels of organic acids, flavonoids, and alkaloids, consistent with their total polyphenol and total flavonoid content. The differences in the aforementioned metabolic features can be attributed to varietal characteristics as well as other factors such as growing region, growth conditions, irrigation, and fertilization.

### 2.4. Antilipidemic Effects of CPFD

#### 2.4.1. CPFD Alleviated OA-Induced Steatosis in HepG2

**Figure 5:**
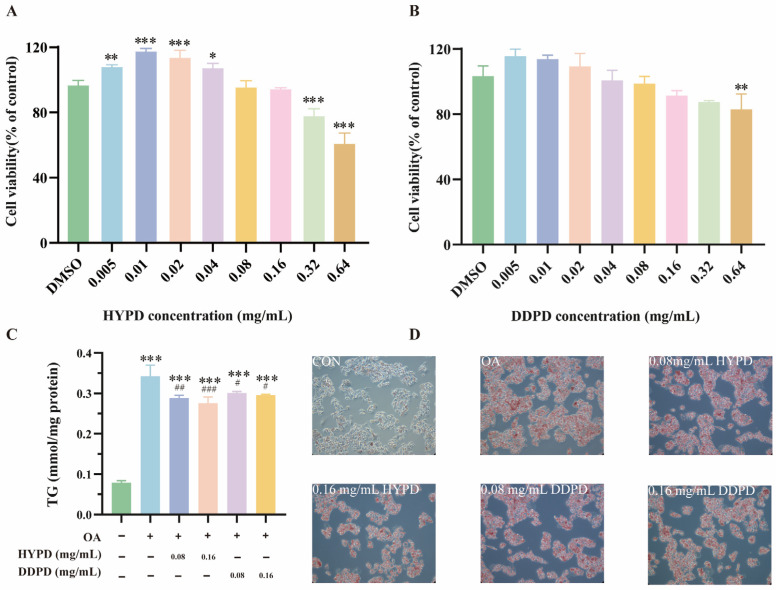
CPFD alleviated OA-induced steatosis in HepG2. (**A**) The effects of HYPD on cell viability in HepG2. (**B**) The effects of DDPD on cell viability in HepG2. (**C**) The effects of CPFDs on TG. (**D**) The morphology of stained intracellular lipid droplets by ORO staining at 200× magnification. Error bars are expressed as means ± SD. The significance levels were denoted as * *p* < 0.05, ** *p* < 0.01, and *** *p* < 0.001. Additionally, # *p* < 0.05, ## *p* < 0.01, and ### *p* < 0.001 were used. OA, Oleic acid; DMSO, Dimethyl sulfoxide; CPFD, citrus physiological premature fruit drop; DDPD, *Citrus aurantium* L. ‘*Daidai*’ physiological premature fruit drop; HYPD, *Citrus aurantium* ‘*Changshan-huyou*’ physiological premature fruit drop; TG, triglyceride.

The human liver cancer cell line, HepG2, is commonly used to simulate liver steatosis induced by obesity [21]. This study utilized the 3-(4,5-Dimethylthiazol-2-yl)-2,5-diphenyltetrazolium bromide (MTT) assay to assess the effects of various concentrations of HYPD and DDPD on HepG2 cell viability. Furthermore, this method determines the optimal treatment concentration of HYPD and DDPD in HepG2 cells and maximizes their efficacy without compromising cell viability. In this study, HYPD treatment at 0.005 to 0.04 mg/mL did not affect the cell viability of HepG2 after 24 h (Figure 5A). When the HYPD concentration increased to 0.08 mg/mL, the cell viability decreased without significant difference. Nevertheless, cell viability significantly decreased to 82.25% compared to the control group (*p* < 0.001) when the HYPD concentration was increased to 0.32 mg/mL. There was also a significant decrease in cell viability (80.33%) compared to the control group (*p* < 0.01) when the cells were treated with 0.64 mg/mL DDPD (Figure 5B). Therefore, we selected two concentrations, 0.16 mg/mL and 0.32 mg/mL, of HYPD and DDPD for subsequent experiments. The accumulation of lipids in hepatocytes is a hallmark of hepatic steatosis. HepG2 cell lines were cultured in a mixture of 0.5 mM OA and CPFD at different concentrations for 24 h to determine the inhibitory effect of different HYPD and DDPD on OA-induced lipid accumulation in the cells. Figure 5C illustrates that the OA treatment increased the triglyceride (TG) content of HepG2 cell lines compared to the control group, but a dose-dependent inhibitory effect was observed in cells that were treated with HYPD. Nonetheless, DDPD exhibited less impact on the TG of HepG2 cells than HYPD. Furthermore, the Oil Red O (ORO) staining confirmed that HYPD and DDPD reduced OA-induced lipid accumulation dose-dependently (Figure 5D).

#### 2.4.2. CPFD Reduced Lipid Accumulation in 3T3-L1 Adipocytes

**Figure 6:**
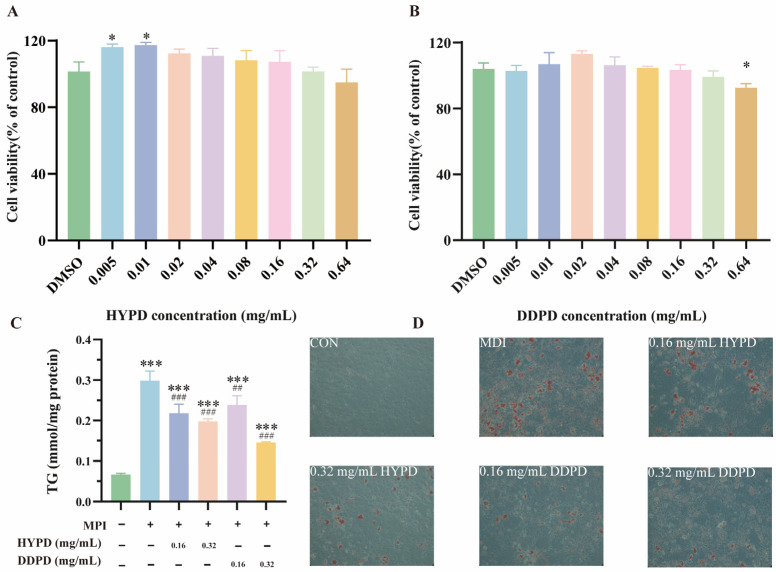
CPFD reduced lipid accumulation in 3T3-L1 adipocytes. (**A**) The effects of HYPD on cell viability in 3T3-L1. (**B**) The effects of DDPD on cell viability in 3T3-L1. (**C**) The effects of CPFDs on TG. (**D**) The morphology of stained intracellular lipid droplets by ORO staining at 200× magnification. Error bars are expressed as means ± SD. The significance levels were denoted as * *p* < 0.05, and *** *p* < 0.001. Additionally, ## *p* < 0.01, and ### *p* < 0.001 were used. DMSO, dimethyl sulfoxide; CPFD, citrus physiological premature fruit drop; DDPD, *Citrus aurantium* L. ‘*Daidai*’ physiological premature fruit drop; HYPD, *Citrus aurantium* ‘*Changshan-huyou*’ physiological premature fruit drop; TG, triglyceride. MDI (methylisobutylxanthine, dexamethasone, insulin; differentiated media).

The 3T3-L1 cell line is one of the most commonly used cellular systems to study adipogenesis. When cultured in the presence of differentiation inducers, these cells exhibit characteristics of mature adipocytes in metabolism and lipid accumulation [22]. In the present study, the effect of CPFD (0.005–0.64 mg/mL) on the survival of 3T3-L1 cells were assessed via MTT assay to determine the non-toxic concentrations of HYPD and DDPD on undifferentiated 3T3-L1 pre-adipocytes. At 0.64 mg/mL, HYPD slightly decreased the cell viability, but the DDPD was significantly different from the control group (*p* < 0.005) (Figure 6A,B). The 3T3-L1 pre-adipocytes differentiated within eight days when treated with 0.16 and 0.32 mg/mL HYPD or DDPD, as described in the methodology section. The results revealed that intracellular TG accumulation increased by 314% in differentiated cells compared to undifferentiated cells. It is worth noting that there was a significant dose-dependent decrease in TG content with increasing concentrations of CPFD. Adding 0.64 mg/mL of HYPD and DDPD decreased TG content by 29.06% and 48.73%, respectively, relative to the differentiated cells (Figure 6C). These findings suggest the superior inhibitory effect of DDPD against lipid accumulation in 3T3-L1 pre-adipocytes. In addition, ORO staining confirmed the dose-dependent reduction in lipid accumulation in 3T3-L1 preadipocytes by HYPD and DDPD (Figure 6D).

## 3. Discussion

Excessive ROS are closely associated with lipid accumulation; thus, reducing cellular ROS levels and inhibiting adipocyte differentiation are critical preventive strategies for alleviating obesity [23]. In this study, CPFD samples were richer in bioactive components and had stronger antioxidant activity than mature fruit of five citrus varieties, which aligned with previous reports [24]. Furthermore, HYPD and DDPD samples were further investigated due to their highly abundant plant bioactive components and prominent antioxidant activities. Based on the untargeted metabolomic analysis of differential metabolites, HYPD recorded more terpenoids and lipids than DDPD. Meanwhile, the antilipidemic in vitro experiments demonstrated that HYPD was superior in inhibiting OA-induced HepG2 fat deposition, while DDPD performed better in the adipogenesis suppression of 3T3-L1 cells.

Citrus plants are rich in various bioactive substances, including carotenoids, flavonoids, limonoids, volatile oils, and coumarins. Several factors, including genetics, ripening stage, and different fruit parts, influence the accumulation of bioactive substances in citrus fruits. Moreover, the composition of citrus fruits varies between species. Untargeted metabolomics primarily relies on LC-MS/MS without pre-selecting or screening specific metabolites but rather aims to explore all possible metabolites present in a sample to obtain comprehensive and integrated metabolic information. In the untargeted metabolomics analysis of differential metabolites, several common compounds were identified in all citrus varieties in the present study, including synephrine, limonene-1,2-epoxide, artemetin, limonoate d-ring-lactone, syringetin 3-glucoside, neohesperidin, diosmetin, and (*R*)-meranzin. Synephrine is a widely occurring alkaloid in citrus fruits, particularly in bitter orange *Citrus aurantium*. This compound exerts a similar effect to adrenaline, such as increasing metabolic rate, promoting energy expenditure, and fat oxidation [25]. Similarly, in this study, DDPD exhibited higher levels of synephrine compared to HYPD. High levels of meranzin were also found in the citrus varieties in this study, which was also reportedly abundant in bitter orange essential oil [26]. This coumarin compound is commonly found in citrus peel [27]. It is noteworthy that among the differential metabolites between HYPD and DDPD, we found that HYPD had a higher abundance of lipids compared to DDPD. However, DDPD accumulated more key products of diacylglycerol, such as PC (16:0/0:0), PC (15:0/0:0), PC (18:1/0:0), PE (16:0/0:0), PE (18:1(9Z)/0:0), MGDG (18:3/18:3), PC (18:2/0:0), and PE (18:2/0:0), following the classical glycerophospholipid biosynthetic pathway, which might contribute to a higher content of essential oil in mature fruits. Interestingly, in another study, we found that the essential oil yields of mature *Citrus aurantium* L. and *Citrus. Changshan huyou*. *B. Chang* were 2.28% and 0.95%, respectively, further supporting the speculation based on the above result [28].

Plant bioactive compounds improve health and prevent chronic diseases in vitro and in vivo by alleviating metabolic disorders, inflammation, and oxidative stress [29,30]. These studies suggest that plant-derived bioactive compounds offer new avenues for developing dietary strategies to prevent various diseases [31]. Hepatic steatosis refers to the excessive accumulation of lipids in the liver, particularly neutral lipid buildup such as TG within lipid droplets (LDs) in hepatocytes [32]. The OA is commonly used to induce hepatic steatosis in HepG2 cells [33]. Studies have investigated the impacts of accelerating TG breakdown in reducing hepatocyte lipid accumulation [34]. In this study, HYPD and DDPD extracts significantly reduced TG levels, indicating their potential antilipidemic effect. Notably, HYPD performed better than DDPD, possibly attributed to the higher terpenoid content in the former than in the latter [35]. Furthermore, neohesperidin exhibited higher expression in HYPD. An earlier study reported that neohesperidin could exert hypolipidemic effects by activating the AMP-activated protein kinase (AMPK) pathway and regulating target genes including Stearoyl-CoA desaturase-1(SCD-1), Fatty acid synthase (FAS), and Acyl-CoA oxidase (ACOX) [36].

Lipogenesis is the process where immature pre-adipocytes differentiate into mature adipocytes, accumulating lipids as fat droplets [37]. Differentiating pre-adipocytes into adipocytes increases intracellular lipid accumulation [38]. The results of this study provided evidence for the efficacy of HYPD and DDPD in attenuating lipogenesis and lipid accumulation in 3T3-L1 adipocytes, with DDPD demonstrating superior effects compared to HYPD. Furthermore, the HYPD and DDPD treatments significantly reduce the accumulation of TG compared to untreated differentiated cells, suggesting potential anti-obesity effects by inhibiting lipid accumulation. At a high dose (0.32 mg/mL), HYPD and DDPD exhibited anti-lipogenesis effects without cytotoxicity on pre-adipocytes and differentiated 3T3-L1 cells. Therefore, HYPD and DDPD could inhibit TG accumulation and reduce lipogenesis without cellular toxicity. A previous study also demonstrated that immature *Citrus sunki HORT. exTANAKA* peel extract increased the phosphorylation of AMPK and acetyl-CoA carboxylase (ACC) in mature 3T3-L1 adipocytes, promoted fatty acid *β*-oxidation, and enhanced lipolysis through the phosphorylation of cAMP-dependent protein kinase (PKA) and hormone-sensitive lipase (HSL) [39]. Synephrine is a naturally occurring alkaloid that is widely used in weight loss due to its low toxicity and excellent fat oxidation effects [40]. The high content of synephrine in DDPD is also likely an important reason for DDPD’s superior ability to inhibit lipid accumulation in 3T3-L1 adipocytes compared to HYDP. In summary, HYPD and DDPD exhibit promising antilipidemic effects in our in vitro cell experiments. However, future studies should investigate the mechanisms underlying the weight-reducing and antilipidemic effects of HYPD and DDPD by incorporating animal models.

## 4. Materials and Methods

### 4.1. Materials

*Citrus aurantium* ‘*Changshan-huyou*’ physiological premature fruit drops and mature fruit drops were obtained from Quzhou, Zhejiang Province, China. *Citrus aurantium* L. ‘*Daidai*’ physiological premature fruit drops and mature fruit drops were obtained from Lianyuan, Hunan Province, China. *Citrus reticulate* ‘*Blanco*’ physiological premature fruit drops and mature fruit drops were obtained from Meishan, Sichuan Province, China. *Citrus reticulata* cv. *Suavissima Ougan* physiological premature fruit drops and mature fruit drops were obtained from Lianyuan, Hunan Province, China. *Citrus maxima* (*Burm.*) *Merr.* cv*. Jiangyong Yu* physiological premature fruit drops and mature fruit drops were obtained from Jiangyong, Hunan Province, China. In this study, citrus physiological premature fruit drops were collected between June and July, and citrus fruits with a diameter between 2.0 and 3.0 cm were selected as experimental materials.

### 4.2. Reagents

Folin–Ciocalteu reagent, trolox (GA, >98%), rutin (GA, >98%), and gallic acid (GA, >98%) were purchased from Chengdu Must Biotechnology Co., Ltd. (Chengdu, China). Methanol and isopropanol were obtained from China National Pharmaceutical Group Chemical Reagent Co., Ltd. (Shanghai, China). MTT, dimethyl sulfoxide (cell culture grade), and bovine serum albumin (BSA) were purchased from Beijing Solarbio Science & Technology Co., Ltd. (Beijing, China). Dexamethasone (Dex), oleic acid, and 3-isobutyl-1-methylxanthine (IBMX) were purchased from Sigma-Aldrich (St. Louis, MO, USA). Fetal bovine serum (FBS), calf serum (CS), phosphate-buffered saline (PBS), trypsin, penicillin-streptomycin (PS), and high-glucose Dulbecco’s modified Eagle’s medium (DMEM) were purchased from Gibco (Rockville, MD, USA).

### 4.3. CPFD Samples and Extracts Preparation

In this study, citrus fruit were subjected to drying and grinding processes. The resulting material was sieved using a 60-mesh screen. Dry citrus fruit samples (1 g) were placed in beakers and mixed thoroughly with 10 mL of 80% methanol. The mixture was subjected to ultrasonic extraction at 35 °C and 40% power for 40 min, followed by centrifugation at 5000 rpm for 15 min. The supernatant was collected and stored at −20 °C for the determination of total phenols, total flavonoids, flavonoids, and antioxidant analysis. The freeze-dried powder of different CPFDs was stored in a cool, dry place until use.

### 4.4. Determinations of Phytochemical Composition

The determination of polyphenol content in citrus fruit samples was conducted using the Folin–Ciocalteu colorimetric method with gallic acid as the standard [41]. Total flavonoid content was determined by the aluminum chloride–sodium nitrite colorimetric method with rutin as the standard [42].

### 4.5. In Vitro Antioxidant Activity Assays of Citrus Fruit Extract

The antioxidant capacity of citrus fruit extract was evaluated using in vitro antioxidant assays: DPPH radical scavenging assay, ABTS radical scavenging assay, and FRAP assay, following the manufacturer guidelines (Suzhou KeMing Biotechnology Co., Ltd., Suzhou, Jiangsu, China). Trolox, a known antioxidant compound, was used as the reference standard in these assays, and the results were expressed in terms of Trolox equivalent (TE) in µmol/mL. Firstly, all fruit extracts were diluted and used in the DPPH, ABTS, and FRAP assays. Subsequently, the absorbance values were used to calculate the antioxidant capacity based on the corresponding Trolox standard curve, and the results were expressed as µmol/mL TE.

### 4.6. Untargeted Metabolomic Analysis of CPFD

Metabolomic analysis of CPFD was conducted using ultra-high-performance liquid chromatography coupled with a quadrupole-Orbitrap hybrid mass spectrometry (UHPLC-QExactive) system (Thermo Fisher Scientific, Waltham, MA, USA). The CPFD powder was prepared by ultrasonication (5 °C, 40 kHz, 30 min) using an extraction solvent (methanol:water = 4:1, v:v). The resultant solution was filtered using a 0.22 µm membrane filter (Waters, Milford, MA, USA) before analysis. Liquid chromatography was conducted using the ACQUITY UPLC HSS T3 column (100 mm × 2.1 mm i.d., 1.8 µm) (Waters Corporation, Milford, MA, USA). The sample injection volume was set at 2 μL, and the column temperature was maintained at 40 °C. Mass spectrometric signals of the samples were acquired in positive and negative ionization modes. Subsequently, the obtained raw data were preprocessed using Progenesis QI 2.3 software (Nonlinear Dynamics, Waters, USA). The preprocessing steps primarily included filtering, deconvolution, alignment, and normalization. Database searches, conducted using KEGG (http://www.genome.jp/kegg/) (accessed on 7 May 2023), were used to summarize the differential metabolites between the two groups. Metabolite enrichment and pathway analysis were performed to map them onto their respective biochemical pathways. Data analysis was conducted on the online platform of Majorbio Cloud Platform (www.majorbio.com (accessed on 7 May 2023)).

### 4.7. Cell Culture

The hepatocellular carcinoma cell line HepG2 and murine fibroblast-derived pre-adipocytes (acquired from Shanghai Fuheng Biological Technology Co., Ltd. (Shanghai, China)) were cultured in DMEM medium supplemented with 10% FBS and 1% PS under a humidified atmosphere of 95% air and 5% CO_2_ at 37 °C. The HepG2 cells were utilized to investigate the inhibitory effects of CPFD on hepatic lipid accumulation, while the 3T3-L1 cells were employed to evaluate the impact of CPFD on differentiated adipocytes.

### 4.8. Cell Viability Assay

HepG2 and 3T3-L1 cells were seeded into a 96-well plate and incubated for 24 h to assess the impacts of HYPD and DDPD on cell viability. Subsequently, the cells were fixed in MTT reagent, and the absorbance was measured at 490 nm using a microplate reader (Tecan, Männedorf, Switzerland).

### 4.9. OA-Induced Steatosis in HepG2 Cells

To detect the effects of CPFD extracts on OA-induced HepG2 cells, HepG2 cells were incubated with DMEM medium containing 0.5 mM OA and CPFD extracts for 24 h.

### 4.10. Differentiation of 3T3-L1 Cells

After seeding 3T3-L1 cells onto culture plates, the cells were cultured in CS medium until 100% confluence was reached. Subsequently, the induction of differentiation was initiated by culturing the cells for two days in 10% CS medium. Next, the cells were cultured for three days in a differentiation medium containing 10% FBS, 1% PS, 1 µM Dex, 10 µg/mL insulin, and 0.5 mM IBMX. Following this, the cells were further cultured for three days in a medium consisting of 10% FBS, 1% PS, and 10 µg/mL insulin. Finally, the cells were cultured for two days in a medium containing 10% FBS and 1% PS. CPFD extracts at different concentrations were simultaneously added to the culture medium at each time point.

### 4.11. Oil Red O Staining

ORO staining was used to assess the cellular morphology in HepG2 and 3T3-L1 cell lines. The staining procedure was conducted using the ORO staining kit (Solaibao Technology Co., Ltd., Beijing, China), following the manufacturer’s guidelines. The cells were washed twice with PBS and fixed using the ORO fixative solution at room temperature. The fixative solution was vertically discarded, and 60% isopropanol solution was added to wash away residual fixative. After gentle agitation, the 60% isopropanol solution was discarded, and freshly prepared ORO working solution was added and incubated at room temperature in the dark for 30 min. The ORO working solution was discarded and rinsed vertically with distilled water 3–4 times to remove residual ORO working solution. Finally, the stained cells were visualized using an inverted microscope (Nikon, Tokyo, Japan).

### 4.12. Cellular Triglyceride Content

After drug treatment, HepG2 and 3T3-L1 cells were washed twice with ice-cold PBS, lysed with lysis buffer, and centrifuged at room temperature at a speed of 2000 rpm for 5 min. The supernatant was collected, and the determination of TG content was conducted strictly according to the instructions provided by the manufacturer of the TG assay kit (Beijing Pulai Gene Technology Co., Ltd., Beijing, China). The cellular protein content was determined using the Bicinchoninic acid protein assay (Shanghai Biyun Tian Biotechnology Co., Ltd., Shanghai, China). The results were expressed as the content of TG per gram of total cellular protein, in units of mmol/g protein.

### 4.13. Statistical Analysis

To compare differences among different samples, we conducted statistical analyses using SPSS 26.0 software. The data were presented as mean ± SD. One-way analysis of variance (ANOVA) was used for comparisons among multiple groups, followed by Tukey tests for analysis. A significance level of *p* < 0.05 was set for all statistical analyses to determine the statistical significance of the results. PCA and PLS-DA were carried out using the freely available Majorbio Cloud Platform (www.majorbio.com) (accessed on 7 May 2023). The heatmap was generated by the freely available Wekemo Bioinclound Platform (https://www.bioincloud.tech) (accessed on 30 November 2023).

## 5. Conclusions

In conclusion, our study elucidates that CPFDs exhibit a profusion of phytochemical constituents and excellent antioxidant activity. Moreover, we found significant differences in organic acids and lipids between HYPD and DDPD. Additionally, HYPD and DDPD remarkably limited excessive fat accumulation in HepG2 and 3T3-L1 cells, and the bioactive compounds present may be responsible for their antioxidant and antilipidemic effects. Therefore, our findings provide alternative utilization channels for HYPD and DDPD. However, further research is needed to identify the key bioactive compounds responsible for the antioxidant and antilipidemic effects in DDPD and DDPD and their potential mechanisms.

## Figures and Tables

**Table 1:** Differential metabolites expression (Fragmentation Score > 50) in DDPD and HYPD.

No	VIP	Rt/min	*m*/*z*	Mode	CAS	Identification	Formula	Classification	Regulate
1	2.4610	2.250766667	307.1760342	pos	-	Feruloylagmatine	C_15_H_22_N_4_O_3_	Others	down
2	2.1676	4.89415	227.0697288	pos	1143-38-0	Anthralin	C_14_H_10_O_3_	Others	down
3	2.0770	6.091066667	194.1173346	pos	89-31-6	Salsoline	C_11_H_15_NO_2_	Alkaloids	down
4	2.0330	3.8725	138.0911662	pos	51-67-2	Tyramine	C_8_H_11_NO	Others	down
5	2.0154	7.645216667	163.0386991	pos	939-19-5	3-Hydroxycoumarin	C_9_H_6_O_3_	Phenylpropanoids	down
6	1.9001	1.9989	134.0962301	pos	-	2-Methylindoline	C_9_H_11_N	Others	up
7	1.8681	3.3298	138.0911105	pos	-	1-(*p*-Hydroxyphenyl) ethylamine	C_8_H_11_NO	Others	down
8	1.8654	7.123033333	193.0494018	pos	776-86-3	Isoscopoletin	C_10_H_8_O_4_	Alkaloids	down
9	1.7830	6.108966667	205.194699	pos	-	2-Isopropenyl-4a,8-dimethyl-1,2,3,4,4a,5,6,7-octahydronaphthalene	C_15_H_24_	Terpenes	down
10	1.7008	4.309816667	308.2213803	pos	148-32-3	Amprotropine	C_18_H_29_NO_3_	Alkaloids	down
11	1.6769	2.36035	226.0704863	pos	-	4-Amino-4-deoxychorismic acid	C_10_H_11_NO_5_	Organic acid	up
12	1.6710	4.109983333	593.1852356	pos	-	Buddleoflavonoloside	C_28_H_32_O_14_	Flavonoids	up
13	1.6570	1.566566667	152.1066258	pos	370-98-9	*N*-Methyltyramine	C_9_H_13_NO	Others	up
14	1.6496	7.9102	434.2884372	pos	-	*N*-arachidonoyl glutamic acid	C_25_H_39_NO_5_	Organic acid	up
15	1.6157	3.963116667	213.148295	pos	172104-03-9	Putaminoxin	C_12_H_20_O_3_	Others	down
16	1.6154	1.78215	242.0653613	pos	7724-78-9	2,3-dihydroxybenzoylserine	C_10_H_11_NO_6_	Others	up
17	1.6147	2.415333333	140.0339558	pos	98-95-3	4-Nitrophenol	C_6_H_5_NO_3_	Others	up
18	1.6026	4.439366667	134.0599624	pos	59-48-3	Oxindole	C_8_H_7_NO	Others	down
19	1.5979	3.528816667	141.0907633	pos	72010-18-5	2-Propyl-2,4-pentadienoic acid	C_8_H_12_O_2_	Lipids	down
20	1.5921	5.166566667	169.1220375	pos	-	8-Hydroxycarvotanacetone	C_10_H_16_O_2_	Terpenes	down
21	1.5477	9.551733333	221.1895876	pos	1139-30-6	Caryophyllene epoxide	C_15_H_24_O	Lipids	down
22	1.5409	2.214966667	170.0807431	pos	-	Pyridoxine	C_8_H_11_NO_3_	Others	up
23	1.5395	5.323516667	235.1688016	pos	-	Alcyopterosins O	C_15_H_22_O_2_	Terpenes	down
24	1.5217	5.622416667	152.1067485	pos	104-09-6	(4-Methylphenyl) acetaldehyde	C_9_H_10_O	Others	down
25	1.4640	8.133916667	136.0755284	pos	103-81-1	2-Phenylacetamide	C_8_H_9_NO	Others	down
26	1.4552	2.58045	222.0756095	pos	-	6-Hydroxyindolelactate	C_11_H_11_NO_4_	Others	up
27	1.4246	2.09015	132.0806876	pos	83-34-1	3-Methylindole	C_9_H_9_N	Others	down
28	1.4186	6.86395	313.2354406	neg	125356-86-7	Leukotoxin diol	C_18_H_34_O_4_	Lipids	down
29	1.4134	4.475616667	153.1271494	pos	1195-92-2	Limonene-1,2-epoxide	C_10_H_16_O	Others	down
30	1.4014	2.342383333	287.1384126	pos	13640-28-3	Pilosine	C_16_H_18_N_2_O_3_	Others	up
31	1.3877	3.222066667	290.0654927	pos	136945-65-8	Dianthramine	C_14_H_11_NO_6_	Alkaloids	up
32	1.3826	1.890766667	135.0803928	pos	-	4-Methoxystyrene	C_9_H_10_O	Others	down
33	1.3678	3.2382	515.1144208	neg	30964-13-7	1,3-Dicaffeoylquinic acid	C_25_H_24_O_12_	Others	up
34	1.3282	1.24845	154.0493069	pos	548-93-6	3-Hydroxyanthranilic acid	C_7_H_7_NO_3_	Others	up
35	1.3248	1.11365	150.0909367	pos	-	Synephrine	C_9_H_13_NO_2_	Alkaloids	up
36	1.3156	1.057266667	298.1280128	pos	-	Phenethylamine glucuronide	C_14_H_19_NO_6_	Others	down
37	1.2997	9.073533333	411.3250925	pos	114020-59-6	(6*α*,22*E*)-6-Hydroxy-4,7,22-ergostatrien-3-1	C_28_H_42_O_2_	Lipids	down
38	1.2961	2.09015	189.1384483	pos	-	*N*, *N*-Dimethyltryptamine	C_12_H_16_N_2_	Alkaloids	down
39	1.2921	8.676833333	380.3153089	pos	-	*N*-linoleoyl valine	C_23_H_41_NO_3_	Organic acid	down
40	1.2772	8.154433333	184.0730015	pos	107-73-3	Phosphocholine	C_5_H_14_NO_4_P	Others	up
41	1.2761	8.536833333	366.2986914	pos	-	Dihydroceramide C2	C_20_H_41_NO_3_	Lipids	down
42	1.2722	7.951316667	205.1947673	pos	-	(−)-*α*-Cedrene	C_15_H_24_	Lipids	down
43	1.2708	2.034916667	114.0913457	pos	-	*N*-Acetylpyrrolidine	C_6_H_11_NO	Others	up
44	1.2635	4.89415	261.1113648	pos	-	(*R*)-Meranzin	C_15_H_16_O_4_	Phenylpropanoids	up
45	1.2584	2.672183333	234.0755659	pos	-	2-Hydroxy-6-oxo-(2′-aminophenyl)-hexa-2,4-dienoate	C_12_H_11_NO_4_	Others	up
46	1.2571	8.154433333	496.3388062	pos	17364-16-8	PC (16:0/0:0)	C_24_H_50_NO_7_P	Lipids	up
47	1.2396	5.039233333	137.1321248	pos	13466-78-9	(+)-3-Carene	C_10_H_16_	Lipids	up
48	1.2385	5.97185	235.1687792	pos	-	Macrophyllic acid A	C_15_H_22_O_2_	Terpenes	down
49	1.2343	5.3979	144.0805222	pos	91-59-8	2-Naphthylamine	C_10_H_9_N	Others	down
50	1.2258	8.016083333	323.2559911	neg	-	11(*R*)-HEDE	C_20_H_36_O_3_	Lipids	down
51	1.2101	2.617466667	331.1280568	pos	5589-85-5	Miraxanthin-III	C_17_H_18_N_2_O_5_	Others	up
52	1.2098	10.02308333	580.5293196	pos	-	Cer(d16:2(4*E*,6*E*)/20:0(2OH))	C_36_H_69_NO_4_	Lipids	down
53	1.2006	3.58255	433.1120777	pos	-	Genistein 4′-O-glucoside	C_21_H_20_O_10_	Flavonoids	up
54	1.1945	5.686116667	309.2041751	neg	111004-08-1	9(*S*)-HpOTrE	C_18_H_30_O_4_	Lipids	down
55	1.1940	2.4533	347.1229536	pos	5375-64-4	Miraxanthin-V	C_17_H_18_N_2_O_6_	Others	up
56	1.1890	3.944766667	271.0591982	pos	-	4,6,4′-Trihydroxyaurone	C_15_H_10_O_5_	Flavonoids	up
57	1.1814	7.526	133.0606782	pos	70-47-3	L-Asparagine	C_4_H_8_N_2_O_3_	Organic acid	down
58	1.1805	8.676833333	118.0862446	pos	2566-34-9	*N*,2-dimethylalanine	C_5_H_11_NO_2_	Organic acid	down
59	1.1795	7.78705	482.3232773	pos	-	PC (15:0/0:0)	C_23_H_48_NO_7_P	Lipids	up
60	1.1652	4.83305	387.104879	neg	479-90-3	Artemetin	C_20_H_20_O_8_	Flavonoids	down
61	1.1645	8.133916667	138.0911354	pos	104-87-0	P-Tolualdehyde	C_8_H_8_O	Others	down
62	1.1630	4.639183333	489.2113061	pos	1180-71-8	Limonoate D-ring-lactone	C_26_H_32_O_9_	Lipids	down
63	1.1538	6.091066667	219.1738452	pos	37208-05-2	Capsidiol	C_15_H_24_O_2_	Lipids	down
64	1.1516	4.89415	175.0386137	pos	34328-51-3	Artemidinal	C_10_H_6_O_3_	Phenylpropanoids	down
65	1.1503	2.143683333	274.1430781	pos	-	Sanguinine	C_16_H_19_NO_3_	Alkaloids	up
66	1.1446	8.69595	326.304721	pos	111-58-0	Oleoyl Ethanolamide	C_20_H_39_NO_2_	Others	down
67	1.1418	2.690183333	164.0703438	pos	-	5-Hydroxy-3,4-dihydrocarbostyryl	C_9_H_9_NO_2_	Others	down
68	1.1383	3.347783333	509.1282039	pos	-	Syringetin 3-glucoside	C_23_H_24_O_13_	Flavonoids	down
69	1.1359	7.78705	520.3390665	pos	-	PC (18:2/0:0)	C_26_H_50_NO_7_P	Lipids	up
70	1.1268	5.003066667	181.1219295	pos	59300-52-6	Norecasantalic acid	C_11_H_16_O_2_	Lipids	down
71	1.1218	3.365666667	247.0957688	pos	23458-02-8	Decursinol	C_14_H_14_O_4_	Phenylpropanoids	up
72	1.1160	0.7165	132.1017484	pos		*β*-Alaninebetaine	C_6_H_13_NO_2_	Amino acids	up
73	1.1116	10.06556667	554.513563	pos	-	Cer(d14:1(4*E*)/20:0(2OH))	C_34_H_67_NO_4_	Lipids	down
74	1.1115	8.1742	454.2919431	pos	53862-35-4	PE (16:0/0:0)	C_21_H_44_NO_7_P	Lipids	up
75	1.1065	3.0777	339.1066468	pos	39015-63-9	*α*-Hydrojuglone 4-*O*-*β*-D-glucoside	C_16_H_18_O_8_	Others	up
76	1.1059	8.457933333	166.0860751	pos	-	2-Propylisonicotinic acid	C_9_H_11_NO_2_	Others	down
77	1.1033	2.842416667	192.0651578	pos	54-16-0	5-Hydroxyindoleacetic acid	C_10_H_9_NO_3_	Others	up
78	1.1012	8.27685	480.3078345	pos	-	PE (18:1(9*Z*)/0:0)	C_23_H_46_NO_7_P	Lipids	up
79	1.0985	1.860016667	353.0843828	neg	327-97-9	Chlorogenic Acid	C_16_H_18_O_9_	Others	up
80	1.0931	9.63365	797.5154884	pos	-	MGDG (18:3/18:3)	C_45_H_74_O_10_	Lipids	up
81	1.0903	8.536833333	132.1017796	pos	1509-34-8	L-Alloisoleucine	C_6_H_13_NO_2_	Organic acid	down
82	1.0854	7.465616667	279.2313458	pos	-	13(*S*)-HODE	C_18_H_32_O_3_	Lipids	down
83	1.0834	7.541266667	293.2095425	neg	-	13(*S*)-HOTrE	C_18_H_30_O_3_	Lipids	down
84	1.0788	6.025033333	263.1263265	neg	178167-75-4	*γ*-CEHC	C_15_H_20_O_4_	Others	down
85	1.0757	7.848633333	442.2946433	pos	-	*N*-(α-Linolenoyl) Tyrosine	C_27_H_39_NO_4_	Lipids	down
86	1.0600	5.603583333	275.1999899	pos	-	6-[5]-ladderane-hexanoic acid	C_18_H_26_O_2_	Lipids	down
87	1.0597	8.27685	522.3547486	pos	-	PC (18:1/0:0)	C_26_H_52_NO_7_P	Lipids	up
88	1.0574	4.785016667	301.0698558	pos	520-34-3	Diosmetin	C_16_H_12_O_6_	Flavonoids	up
89	1.0526	8.594583333	300.2891351	pos	544-31-0	Palmitoyl Ethanolamide	C_18_H_37_NO_2_	Organic acid	down
90	1.0239	3.2759	611.1962817	pos	13241-33-3	Neohesperidin	C_28_H_34_O_15_	Flavonoids	down
91	1.0220	4.584333333	187.0385834	pos	-	Pyrogallin	C_11_H_8_O_4_	Others	up
92	1.0214	7.227266667	285.2043948	neg	42150-38-9	Hexadecanedioic acid	C_16_H_30_O_4_	Lipids	down
93	1.0207	0.68085	160.096405	pos	515-25-3	(−)-Betonicine	C_7_H_13_NO_3_	Organic acid	up
94	1.0060	4.475616667	133.1010276	pos	1195-32-0	4-Isopropenyltoluene	C_10_H_12_	Others	down
95	1.0030	7.828316667	478.292056	pos	-	PE (18:2/0:0)	C_23_H_44_NO_7_P	Lipids	up
96	1.0011	2.672183333	274.106699	pos	1596-67-4	L-Thyronine	C_15_H_15_NO_4_	Organic acid	up
97	1.0010	8.564383333	312.2514222	neg	158305-64-7	*N*-Palmitoyl Glycine	C_18_H_35_NO_3_	Organic acid	down

PC, phosphatidylcholine; PE, Phosphatidylethanolamine; MGDG, monogalactosyldiacylglycerol; HODE, Hydroxyoctadecadienoic acid; HOTrE, Hydroxyoctadecatrienoic acid; up, metabolites are enriched in DDPD; down, metabolites are enriched in HYPD.

## Data Availability

Data are contained within the article.

## Supplementary Materials

The supporting information can be downloaded at: https://www.mdpi.com/article/10.3390/ijms25031876/s1.
