# Supplementary material for: Untargeted Metabolomic Analyses and Antilipidemic Effects of Citrus Physiological Premature Fruit Drop
Source: Int J Mol Sci. 2024 Feb 4;25(3):1876. doi: 10.3390/ijms25031876 (PMC10855584; doi:10.3390/ijms25031876)
Supplement: Supplementary file 1 [file ijms-25-01876-s001.zip › ijms-2832544-supplementary.pdf]

Supplementary materials

Untargeted Metabolomic Analyses and Antilipidemic Effects of Citrus Physiological Premature Fruit Drop

Chao Wang [1,2], Mingfang Peng [1,2], Zhipeng Gao [3], Qi Han [4], Fuhua Fu [1,2], Gaoyang Li [1,2], Donglin Su [1,2], Lvhong Huang [2], Jiajing Guo [2,*] and Yang Shan [1,*]

[1] Longping Branch, College of Biology, Hunan University, Changsha 410125, China
[2] Hunan Agriculture Product Processing Institute, Dongting Laboratory, Hunan Provincial Key Laboratory of Fruits & Vegetables Storage, Processing, Quality and Safety, Hunan Academy of Agricultural Sciences, Changsha 410125, China
[3] Fisheries College, Hunan Agricultural University, Changsha 410128, China
[4] College of Animal Science and Technology, Hunan Agricultural University, Changsha 410128, China
* Correspondence: guojiajing1986@163.com (J.G.); sy6302@sohu.com or sy6302@hnu.edu.cn (Y.S.); Tel.: +86-(0)731-8469-8915 (J.G.); +86-(0)731-8469-1289 (Y.S.)

Total Pages: 2; Total Figures:2

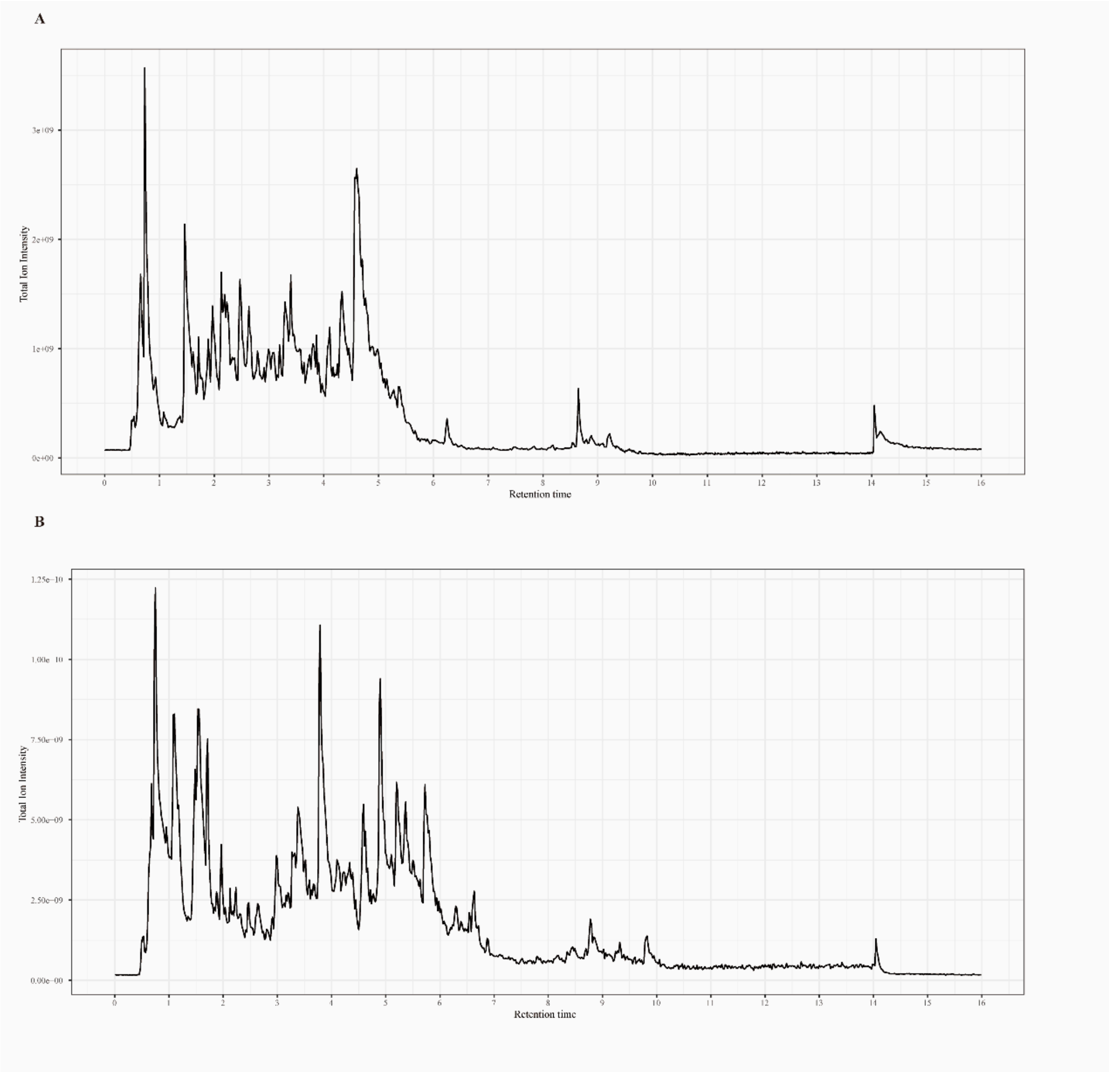

Figure S1. Total ion current (TIC) chromatograms of *Citrus aurantium L. 'Daidai'* physiological premature fruit drop. **(A)** negative mode. **(B)** positive mode.

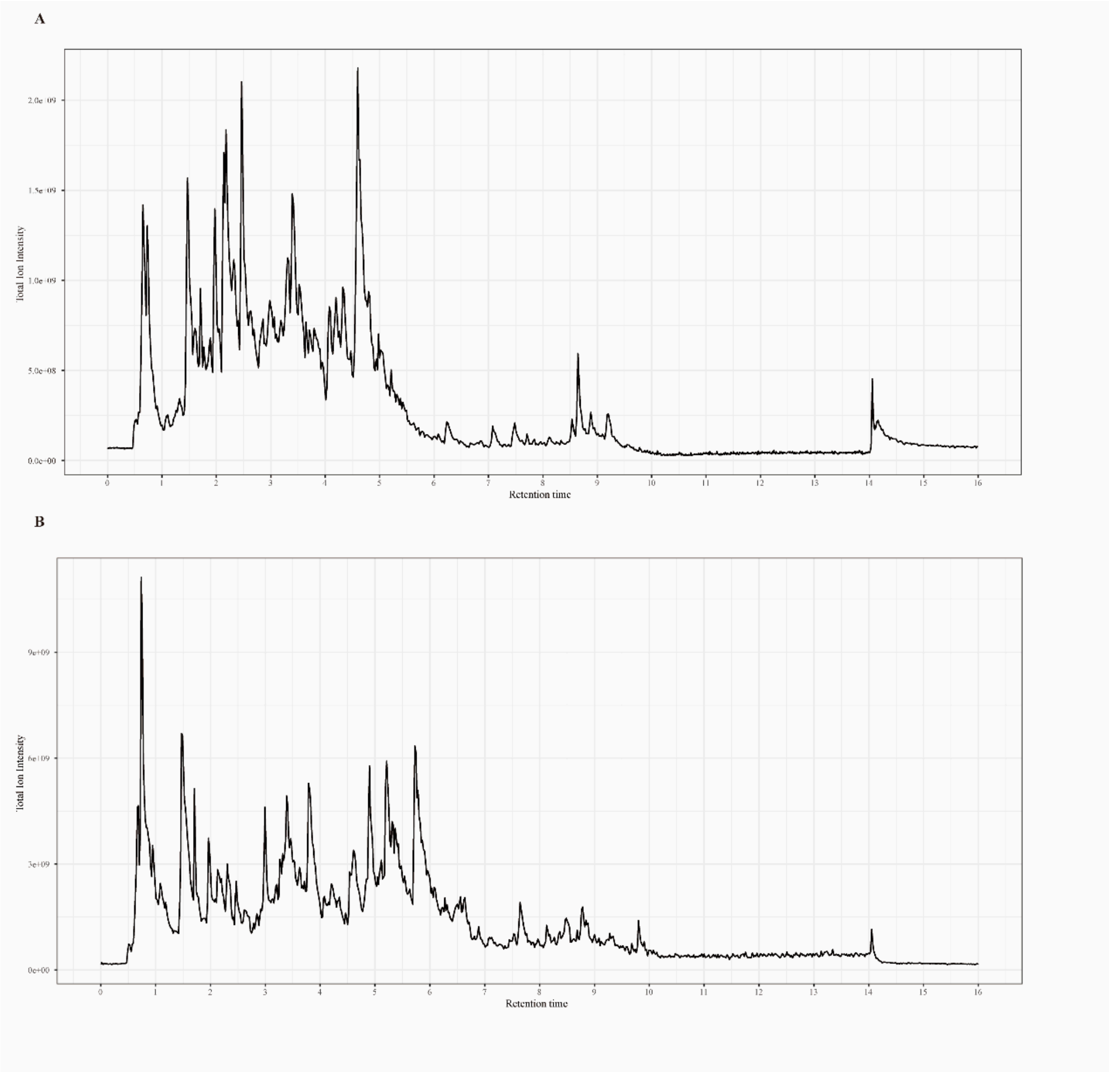

Figure S2. Total ion current chromatograms of *Citrus aurantium 'Changshan-huyou'* physiological premature fruit drop. **(A)** negative mode. **(B)** positive mode.
